# Development of HiBiT-Tagged Recombinant Infectious Bronchitis Coronavirus for Efficient *in vitro* and *in vivo* Viral Quantification

**DOI:** 10.3389/fmicb.2020.02100

**Published:** 2020-08-28

**Authors:** Xiao Ying Liang, Qing Chun Zhu, Jia Qi Liang, Si Ying Liu, Ding Xiang Liu, To Sing Fung

**Affiliations:** ^1^Guangdong Province Key Laboratory of Microbial Signals & Disease Control, Integrative Microbiology Research Centre, South China Agricultural University, Guangzhou, China; ^2^Guangdong Laboratory for Lingnan Modern Agriculture, South China Agricultural University, Guangzhou, China

**Keywords:** coronavirus, recombinant virus, virus replication, luminescence, spike (S) protein, membrane (M) protein

## Abstract

Coronaviruses (CoVs) are enveloped (+) ssRNA viruses of veterinary and medical importance. Because recombinant CoVs with reporter proteins fused with viral proteins are usually non-viable or unstable, a small and quantifiable epitope tag would be beneficial to CoV research. In this study, we integrated the NanoLuc Binary Technology to the reverse genetics of infectious bronchitis virus (IBV), a prototypic gammacoronavirus. The 11-amino-acid HiBiT tag was inserted to the spike (S) or membrane (M) protein, and the recombinant IBVs (rS-HiBiT and rM-HiBiT) were characterized. Compared with the rIBV-p65 control, rS-HiBiT exhibited comparable growth kinetics, whereas rM-HiBiT replicated slightly slower. The levels of HiBiT-tagged S and M proteins in the infected cells or the culture supernatant could be both rapidly (~15 min) and efficiently (30 μL sample volume) determined using the HiBiT luminescence assay. Notably, replication of the HiBiT-tagged IBV could be monitored continuously in an infected chicken embryo, and rS-HiBiT was genetically stable for at least 20 passages. By integrating the HiBiT tagging system with CoV reverse genetics, this new reporter system may facilitate future study of CoV replication and pathogenesis.

## Significance

The ongoing pandemic of coronavirus disease 2019 (COVID-19), caused by a newly emerged zoonotic coronavirus SARS-CoV-2, highlights the importance of coronaviruses (CoVs) as human and animal pathogens. Recombinant CoVs harboring reporter proteins are valuable research tools, but they are usually non-viable or unstable when reporters are directly fused with viral proteins. In this study, we adopted the novel HiBiT tag and inserted it internally to the spike (S) and membrane (M) protein of the gammacoronavirus infectious bronchitis virus (IBV). This strategy enabled us to rapidly, efficiently, and continuously quantify the levels of HiBiT-tagged IBV proteins in biological samples, such as culture supernatant and allantoic fluid of infected chicken embryos. With its small size (11-amino-acid) and quantifiable nature, the HiBiT tag may be a powerful tool adding to CoV reverse genetics, facilitating mechanistic study of CoV replication and pathogenesis when combined with the numerous animal models for human CoVs.

## Introduction

Coronaviruses (CoVs) are important animal pathogens that can cause zoonotic diseases, such as severe acute respiratory syndrome (SARS), Middle East respiratory (MERS) and coronavirus disease 2019 (COVID-19). As enveloped viruses with non-segmented, single-stranded, and positive-sense RNA genomes, CoVs mainly encode four structural proteins: the spike (S), membrane (M), envelope (E), and nucleocapsid (N) protein. The S protein is a large trimeric glycoprotein that mediates receptor binding and membrane fusion during entry (Masters, 2006; Yamada and Liu, 2009). The M protein is the most abundant structural protein that orchestrates virion assembly and provides mechanical support for the virion envelope (Lim and Liu, 2001; Hogue and Machamer, 2008). Although present in limited amounts, the E protein is critical for virion assembly and release, and it also modulates virulence and pathogenesis with its recently identified ion channel activity (Nieto-Torres et al., 2014; To et al., 2017). Finally, the N protein encapsidates the genome in a beads-on-a-string fashion, presumably also contributing to viral RNA transcription and other processes (Baric et al., 1988; Masters, 2006).

To facilitate CoV research, a few reporter proteins have been incorporated into CoV genomes using reverse genetics. There are mainly three approaches. First, a transcription regulatory sequence (TRS) is placed upstream of a reporter gene, and this expression cassette is inserted in the intergenic region of the CoV genome (Shen et al., 2009). Secondly, one or more accessory genes are replaced with a reporter gene (Sola et al., 2003; Wong et al., 2015). Thirdly, a reporter gene is fused with a structural or a non-structural gene (Freeman et al., 2014; V'kovski et al., 2019). In the first two approaches, the expression level of the reporter gene is often affected by the size and sequence composition of the transgene, as well as the location of insertion/replacement in the viral genome. For the third approach, the fusion of the reporter gene may affect the normal function of the viral protein, rendering the recombinant CoVs attenuated or non-viable (Shen et al., 2009; Hagemeijer et al., 2011; Freeman et al., 2014). Even if the insertion/fusion of reporter genes was tolerated, the recombinant CoV might be unstable and the heterologous gene would be rapidly eliminated within several passages (Sola et al., 2003; Shen et al., 2009).

In comparison, short epitope tags are significantly smaller and appear to be more flexible for the labeling of coronavirus proteins. Previously we have recovered recombinant infectious bronchitis coronaviruses (rIBVs) with a FLAG-tag inserted after the S1/S2 cleavage site or at the C-terminus of the S protein (Yamada and Liu, 2009), or with an HA-tag inserted in the N-terminus of nsp12 (Tan et al., 2018). All three rIBVs replicated similarly to the parental control in cell culture (Yamada and Liu, 2009; Tan et al., 2018). Short epitope tags have also been successfully incorporated in other coronaviruses, such as a tetracysteine (TC)-tag in the E protein of MHV (Venkatagopalan et al., 2015), an HA-tag in the nsp15 of MHV (Athmer et al., 2017), a Myc-tag in the ORF3 of porcine epidemic diarrhea virus (PEDV) (Kaewborisuth et al., 2018), and a FLAG-tag in the 7b protein of feline infectious peritonitis (FIPV) (Florek et al., 2017).

NanoLuc (Nluc) is a novel luminescent protein engineered from the luciferase of deep-sea shrimp (*Oplophorus gracilirostris*). Compared with firefly luciferase (Fluc, 61 kDa) or Renilla luciferase (Rluc, 36 kDa), Nluc is significantly smaller (19.1 kDa), while exhibiting higher luminescent intensity (Hall et al., 2012). Recently, the NanoLuc Binary Technology (NanoBiT) was developed, in which Nluc was separated into an 18 kDa large subunit (LgBiT) and an 11-amino acid peptide tag (HiBiT) (Schwinn et al., 2018). The affinity between HiBiT and LgBiT is extremely high, with a dissociation constant (Kd) value as low as 0.7 nM (Schwinn et al., 2018). Due to its small size, the HiBiT tag can be efficiently added to a cellular protein of interest (POI) by CRISPR, and the HiBiT-tagged POI can be easily quantified by luminescence after reconstituted with the LgBiT present in the reaction buffer (Schwinn et al., 2018).

Nluc and NanoBiT have been successfully utilized to generate reporter systems for some viruses. For example, Nluc was fused to the PA protein to generate a reporter influenza A virus, which stably maintained Nluc and replicated with near-native properties in cell culture and mice (Tran et al., 2013). This reporter virus allowed serial observations of viral load and dissemination in the mouse lungs using bioluminescent imaging (Tran et al., 2013). Recombinant viruses bearing Nluc have also been constructed for human immunodeficiency virus-1 (HIV-1) (Astronomo et al., 2016), Dengue virus (Eyre et al., 2017), rotavirus (Kanai et al., 2017), and SARS-CoV (Agostini et al., 2018). In terms of NanoBiT, a recent study has generated recombinant flaviviruses with the HiBiT-tag inserted at the N terminus of NS2 (Tamura et al., 2018). Replicating comparably to the parental viruses, these recombinant viruses were used to screen for antivirals against flaviviruses (Tamura et al., 2018). The NanoBiT system was also adopted to a subviral particle (SVP) system to study the entry of West Nile virus (Sasaki et al., 2018). So far, no other recombinant CoVs harboring the HiBiT-tag has been reported.

In this study, we have constructed two rIBVs with HiBiT-tag inserted to the structural protein S or M, respectively. The rS-HiBiT virus was genetically stable and exhibited comparable growth kinetics as the parental rIBV-p65, whereas rM-HiBiT was less stable and its replication slightly slower. The expression of HiBiT-tagged S and M protein could be determined by HiBiT blotting, and the post-translational modifications were not affected. The protein levels of S and M proteins in the culture supernatant and cell lysates could be rapidly (~15 min) and efficiently (~30 μL sample volume) determined using the HiBiT luminescence assay, providing a convenient surrogate assay for virus growth kinetics. Notably, using the HiBiT-tagged rIBVs, we were also able to monitor virus growth continuously in a single infected embryonated chicken egg. Interestingly, whereas both rS-HiBiT and rM-HiBiT showed comparable *in vivo* infectivity, rS-HiBiT was significantly attenuated in chicken embryos. Taken together, our data establish the advantages of using HiBiT-tagged recombinant viruses to study CoV replication and pathogenesis.

## Results

### Recovery of rIBVs With HiBiT Tag Inserted to the S or M Protein

**Figure 1:**
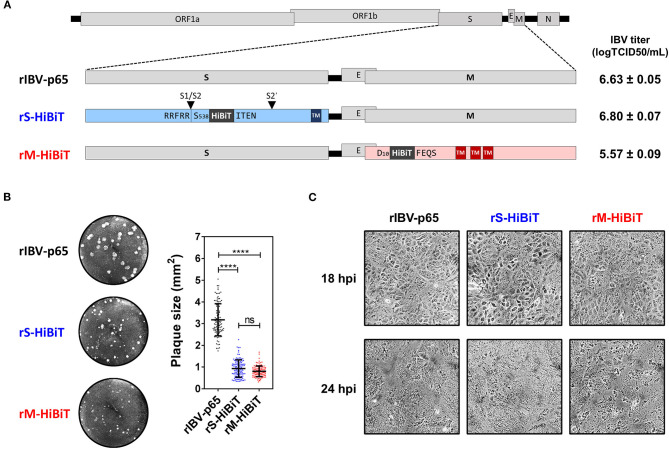
Construction and characterization of HiBiT-tagged rIBVs. **(A)** Schematic diagram showing the genomic structures of rIBVs used in this study. The HiBiT tag was inserted immediately after S538 and D10 in rS-HiBiT and rM-HiBiT, respectively. S1/S2 and S2′ represent the proteolytic cleavage sites in the S protein. Titers of the IBV stocks were shown in the unit of log TCID50 per ml. TM, transmembrane domain. **(B)** Plaque morphologies and plaque areas of the rIBVs. Representative images of the plaque morphologies of the above three rIBVs were shown. The areas of at least 100 plaques were determined and plotted for each rIBV. The experiment was repeated three times with similar results, and the result of one representative experiment is shown. ****p < 0.0001; ns, non-significant. **(C)** Cytopathic effect of Vero cells infected with the rIBVs. Vero cells were infected with rIBV-p65, rS-HiBiT, or rM-HiBiT at a multiplicity of infection (MOI) of ~2. Phase images were captured at 18 and 24 h post-infection (hpi). The experiment was repeated three times with similar results, and the result of one representative experiment is shown.

Previously we have reported the adaptation of the Beaudette strain of IBV to Vero cells (IBV-p65, accession no. DQ001339) (Fang et al., 2005), and the recovery of recombinant IBV-p65 (rIBV-p65) from its cDNA clone (Fang et al., 2007). Using this reverse genetics approach, the coding sequence of the HiBiT tag with a flexible linker (GSSG) flanking on either side was inserted into the IBV structural genes. As shown in Figure 1A, a HiBiT tag was inserted between S538 and I539 of the S protein, immediately after the S1/S2 cleavage site, to generate rS-HiBiT. Similarly, a HiBiT tag was inserted between D10 and F11 of the M protein to generate rM-HiBiT. The parental rIBV-p65 control, rS-HiBiT, and rM-HiBiT were successfully recovered and titers of the virus stock were determined by TCID50 assay. Whereas rIBV-p65 and rS-HiBiT achieved similar high titers, the titer of rM-HiBiT was lower by ~1-log (Figure 1A). All three rIBVs could form plaques in Vero cells (Figure 1B). Whereas the average plaque size of rIBV-p65 was about 3 square millimeters, those of rS-HiBiT and rM-HiBiT were significantly smaller (Figure 1B). When Vero cells were infected at a multiplicity of infection (MOI) of ~2, typical cytopathic effect (CPE) in the form of multinucleated syncytium was observed at comparable levels in cells infected with the three rIBVs (Figure 1C). At 24 h post-infection (hpi), complete cell-cell fusion was observed for all three rIBVs. Taken together, the result suggests that although rS-HiBiT and rM-HiBiT formed smaller plaques, they could infect and cause CPE in Vero cells.

### Determining the Expression of HiBiT-Tagged Proteins

**Figure 2:**
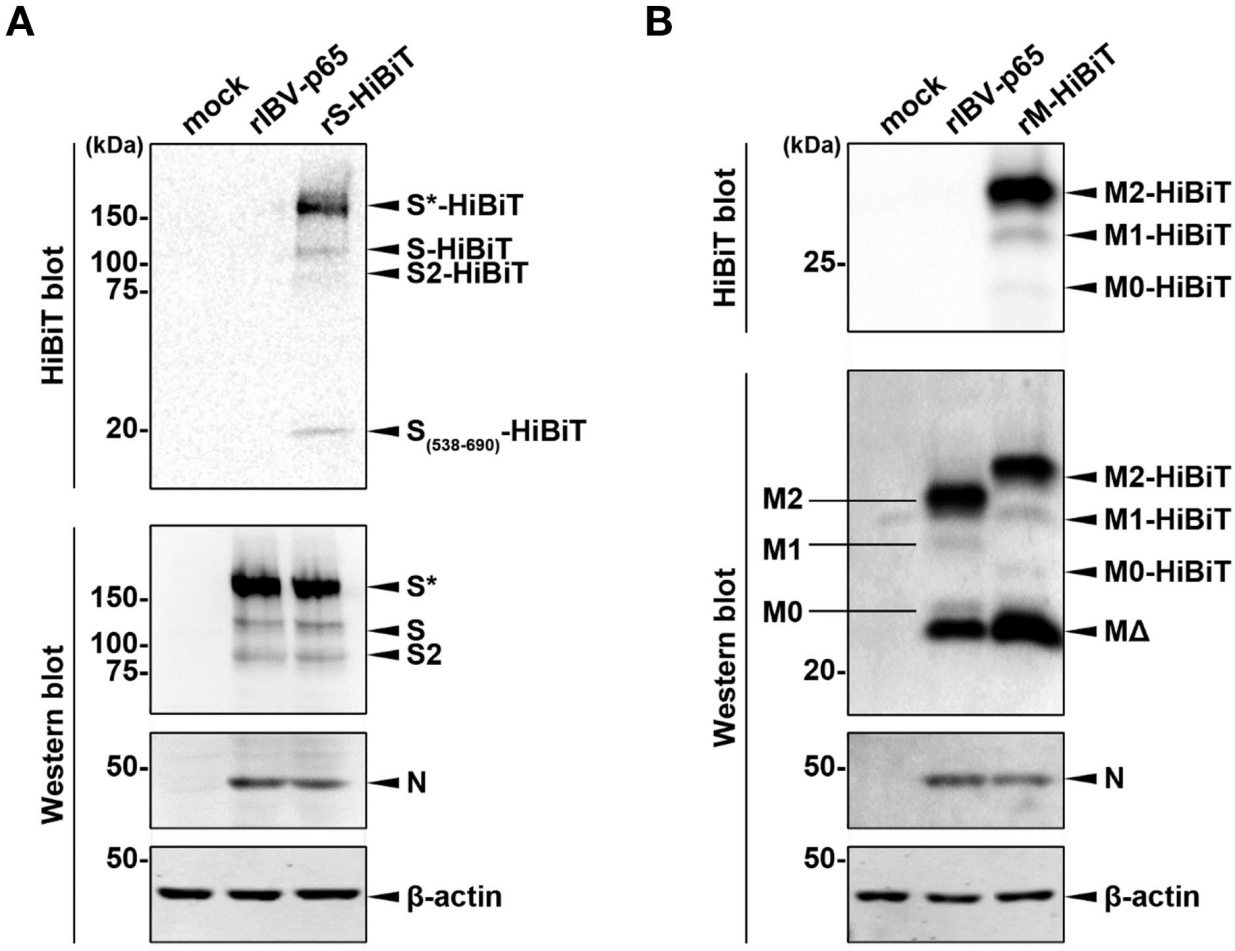
Determining the expression of HiBiT-tagged proteins. **(A)** Determination of S-HiBiT expression in Vero cells infected with rS-HiBiT. Vero cells were infected with rIBV-p65 or rS-HiBiT at MOI ~ 2, or incubated with mock lysate for 18 h. Protein samples were resolved by SDS-PAGE and subjected to HiBiT blot and Western blot using antisera against IBV S2 and N. Beta-actin was used as the loading control. Sizes of the protein ladders in kDa were indicated on the left. The experiment was repeated three times with similar results, and the result of one representative experiment is shown. S*, glycosylated S protein. **(B)** Determination of M-HiBiT expression in Vero cells infected with rM-HiBiT. Vero cells were infected with rIBV-p65 or rM-HiBiT at MOI ~ 2, or incubated with mock lysate for 24 h. Protein samples were resolved by SDS-PAGE and subjected to HiBiT blot as in **(A)**. Western blot was performed using antisera against IBV M and N. Beta-actin was used as the loading control. Sizes of the protein ladders in kDa were indicated on the left. The experiment was repeated three times with similar results, and the result of one representative experiment is shown. M0, unglycosylated M protein; M1, singly glycosylated M protein; M2, dual glycosylated M protein; MΔ, a putative cleavage product of M protein.

To confirm the expression of HiBiT-tagged structural proteins, lysates of cells infected with the three rIBVs at MOI ~ 2 were analyzed by Western blot and HiBiT blot. As shown in Figure 2A, similar levels of S and N protein were detected by Western blot in cells infected with rIBV-p65 or rS-HiBiT. Also, the relative intensities of bands corresponding to the glycosylated full-length S (S^*^), non-glycosylated full-length S, and S2 subunit were comparable, suggesting that insertion of the HiBiT tag did not significantly affect the glycosylation and S1/S2 proteolytic cleavage of IBV S protein in the infected cells. When the same lysates were analyzed by HiBiT blot, HiBiT-tagged S^*^, S, and S2 were all detected in cells infected rS-HiBiT. Additionally, a small band with a molecular weight of roughly 20 kDa was observed. This band represented the peptide fragment S538-R690 of the S protein flanked by the cleavage sites S1/S2 (RRFRR/S_538_) and S2′ (RRRR_690_/S), with a predicted size of 18.9 kDa.

In another experiment, Western blot using IBV M antiserum detected four bands in the lysate of rIBV-p65- and rM-HiBiT-infected cells (Figure 2B). As demonstrated in our previous study, the largest and strongest band (M2) is M protein N-linked glycosylated at both Asn3 and Asn6, whereas the faint bands M1 and M0 corresponded to the mono- and unglycosylated protein, respectively. The strong band (MΔ) smaller than M0 was presumably a cleavage form of the M protein. Due to the insertion of HiBiT tag, M2-HiBiT, M1-HiBiT, and M0-HiBiT all migrated slightly slower than the corresponding non-tagged proteins in the rIBV-p65 control. Interestingly, the M2-HiBiT band was slightly weaker than non-tagged M2, whereas the MΔ band appeared stronger in cells infected with rM-HiBiT than in the rIBV-p65 control. This suggests that the HiBiT-tagged M protein might be more susceptible to cleavage compared to non-tagged M. Despite its abundance in the Western blot, no MΔ band could be observed in the HiBiT blot, whereas M2-HiBiT, M1-HiBiT, and M0-HiBiT were all detectable. Taken together, both the HiBiT-tagged S and M protein were expressed at comparable levels as the non-tagged counterparts, and the proteolytic cleavage and glycosylation were not significantly affected.

### Growth Kinetics of HiBiT-Tagged rIBVs in Culture Cells

**Figure 3:**
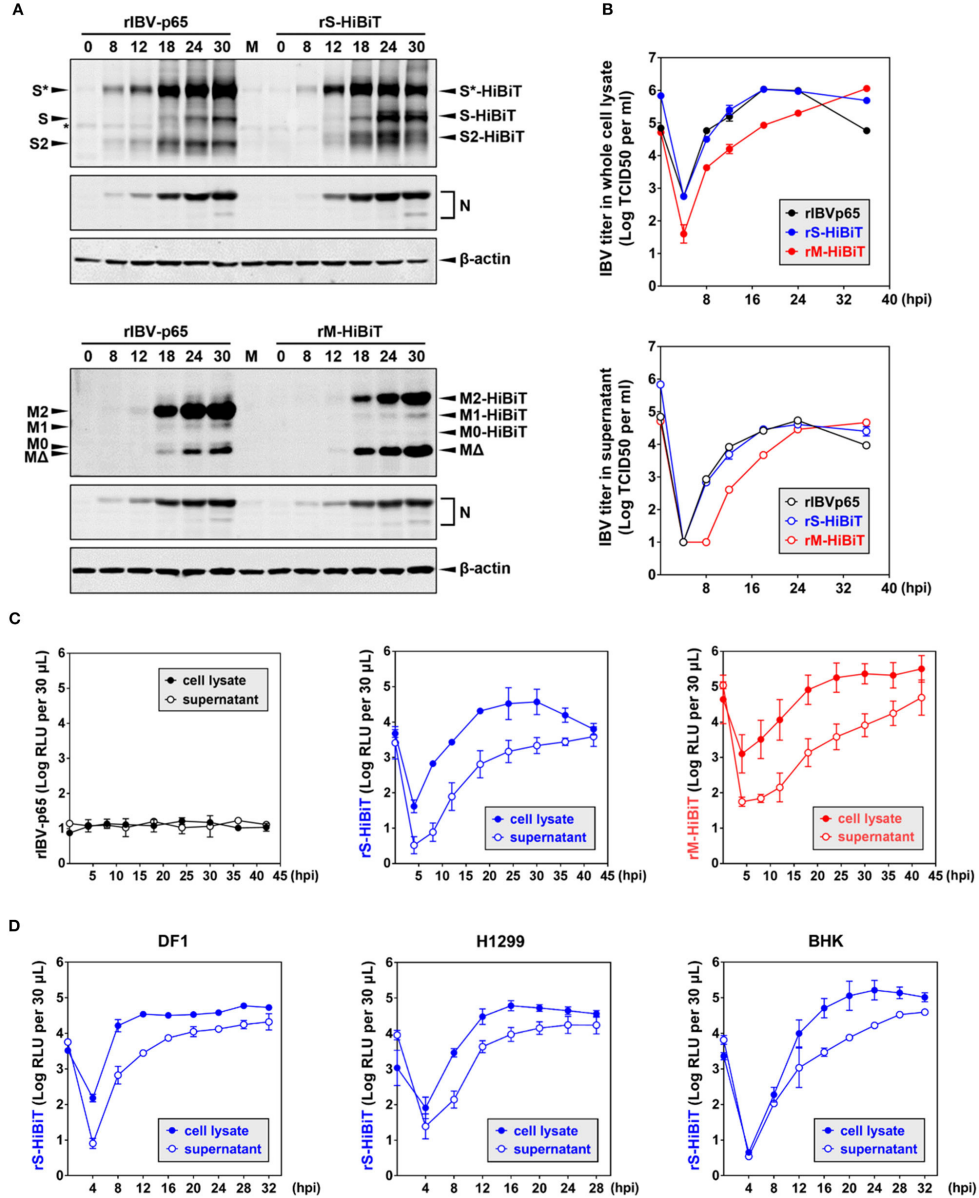
Growth kinetics of HiBiT-tagged rIBVs in culture cells. **(A)** Viral protein synthesis in Vero cells infected with the rIBVs in a time course infection. Vero cells were infected with rIBV-p65, rS-HiBiT, or rM-HiBiT at MOI ~ 2. Cell lysates were harvested by three freeze/thaw cycles at the indicated time points and subjected to SDS-PAGE, followed by Western blot analysis using antisera against IBV S2, M, and N. Beta-actin was used as the loading control. The experiment was repeated three times with similar results, and the result of one representative experiment is shown. **(B)** Growth kinetics of the three rIBVs in Vero cells. Vero cells were infected as in **(A)**. Cell lysate and supernatant samples harvested at the indicated time points were subjected to virus titration using the TCID50 method. IBV titers were expressed in the unit of log TCID50 per ml. The experiment was repeated three times with similar results, and the result of one representative experiment is shown. **(C)** Quantification of HiBiT protein by luminescent assays in rIBV-infected cells. Vero cells were infected as in **(A)**. 30 μL cell lysate or supernatant samples were subjected to luminescent analysis using the Nano-Glo HiBiT lytic detection system. The background (medium only) was subtracted from the measured relative light unit (RLU) values, and luminescent intensities were expressed in the unit of log RLU per 30 μL. The experiment was repeated three times. The average values and standard deviations of the three experiments were shown. **(D)** Quantification of HiBiT protein in other cell lines infected with rS-HiBiT. DF1, H1299, and BHK cells were infected with rIBV-p65 or rS-HiBiT at MOI ~ 2. Quantification of S-HiBiT was done as in **(C)**. The experiment was repeated three times. The average values and standard deviations of the three experiments were shown.

To compare the growth kinetics of the three rIBVs, Vero cells were infected at MOI ~ 2 in a timecourse experiment. As shown in Figure 3A, similar levels of S and N protein were detected for rIBV-p65 and rS-HiBiT of the same time points. At 24 and 30 hpi, the levels of glycosylated S protein were slightly higher in cells infected with rIBV-p65, but the levels of unglycosylated S and S2 subunit were higher in cells infected with rS-HiBiT, suggesting a minor difference in S protein processing at the late stage of IBV infection. On the other hand, similar N protein translation was observed for rIBV-p65 and rM-HiBiT. Throughout the timecourse, the M2 band was weaker and the MΔ band was stronger for rM-HiBiT compared with rIBV-p65.

Virus titers in the whole-cell lysates and supernatants were also compared. As shown in Figure 3B, both rIBV-p65 and rS-HiBiT propagated equally well in Vero cells, with lysate titers peaking at 18 hpi and supernatant titers peaking at 24 hpi. The decrease in virus titers at 36 hpi seemed less significant in cells infected with rS-HiBiT compared with the rIBV-p65 control. In contrast, virus titers in the whole-cell lysates were about 1-log lower at early time points (4–18 hpi) for rM-HiBiT compared with rIBV-p65. The peak titer of rM-HiBiT, comparable to that of rIBV-p65 and rS-HiBiT, was achieved at the very end of the timecourse. In terms of supernatant titer, there was also a delay of 6–8 h for rM-HiBiT, although it eventually reached a similar peak titer as rIBV-p65 and rS-HiBiT. In summary, compared with rIBV-p65, rS-HiBiT replicated similarly and rM-HiBiT replicated slower in cell culture.

We then used the HiBiT assay to determine the growth kinetics of the rIBVs. An extremely low level of basal luminescence was measured for rIBV-p65 throughout the timecourse (Figure 3C). In cells infected with rS-HiBiT, the curve of luminescence in the cell lysate strongly resembles the virus growth curve, peaking at 24–32 hpi followed by a gradual reduction afterward. A very low relative light unit (RLU) value was read at 4 hpi, probably from the small amount of internalized rS-HiBiT virions. As for the rS-HiBiT supernatant luminescence, after an eclipse period during 4–8 hpi, the supernatant RLU value rose rapidly during 12–24 hpi and plateaued afterward. Supernatant RLU values were about 1-log lower than cell lysate RLU values from 4 to 24 hpi, but the two values eventually converged at 42 hpi, possibly due to cell death and the release to intracellular S-HiBiT protein to the supernatant. The luminescence curves of rM-HiBiT were different from those of rS-HiBiT. First, the RLU values of the same timepoint were significantly higher than those of rS-HiBiT, because M protein is more abundant than the S protein. This also resulted in a much higher background reading during the eclipse period for rM-HiBiT. Secondly, after reaching its peak at about 24 hpi, the cell lysate luminescence for rM-HiBiT remained at high levels till the end of the timecourse, consistent with its slower growth kinetics in Figure 3B.

We then determined the replication of rS-HiBiT in three other cell lines previously shown to be susceptible to IBV infection: the chicken embryonic fibroblast cells DF1, the human non-small cell lung carcinoma cells H1299, and the baby hamster kidney cells BHK (Tay et al., 2012). The patterns of the luminescence curves were similar to those of the Vero cells, although the time points for peak luminescence differed: 12 hpi in DF1 cells, 16 hpi in H1299 cells, and 20–24 hpi in BHK cells (Figure 3D). Notably, for DF1 and H1299 cells, the cell lysate luminescence reached similar levels as in Vero cells, but the supernatant luminescence was slightly higher, suggesting that the release of S-HiBiT protein was more efficient. Although RLU values increased slower in the BHK cells, the peak cell lysate luminescence was the highest among the four tested cell lines. Taken together, the growth of rS-HiBiT in cultured cells could be determined using the HiBiT luminescence assay.

### Genetic Stability of HiBiT-Tagged rIBVs

**Figure 4:**
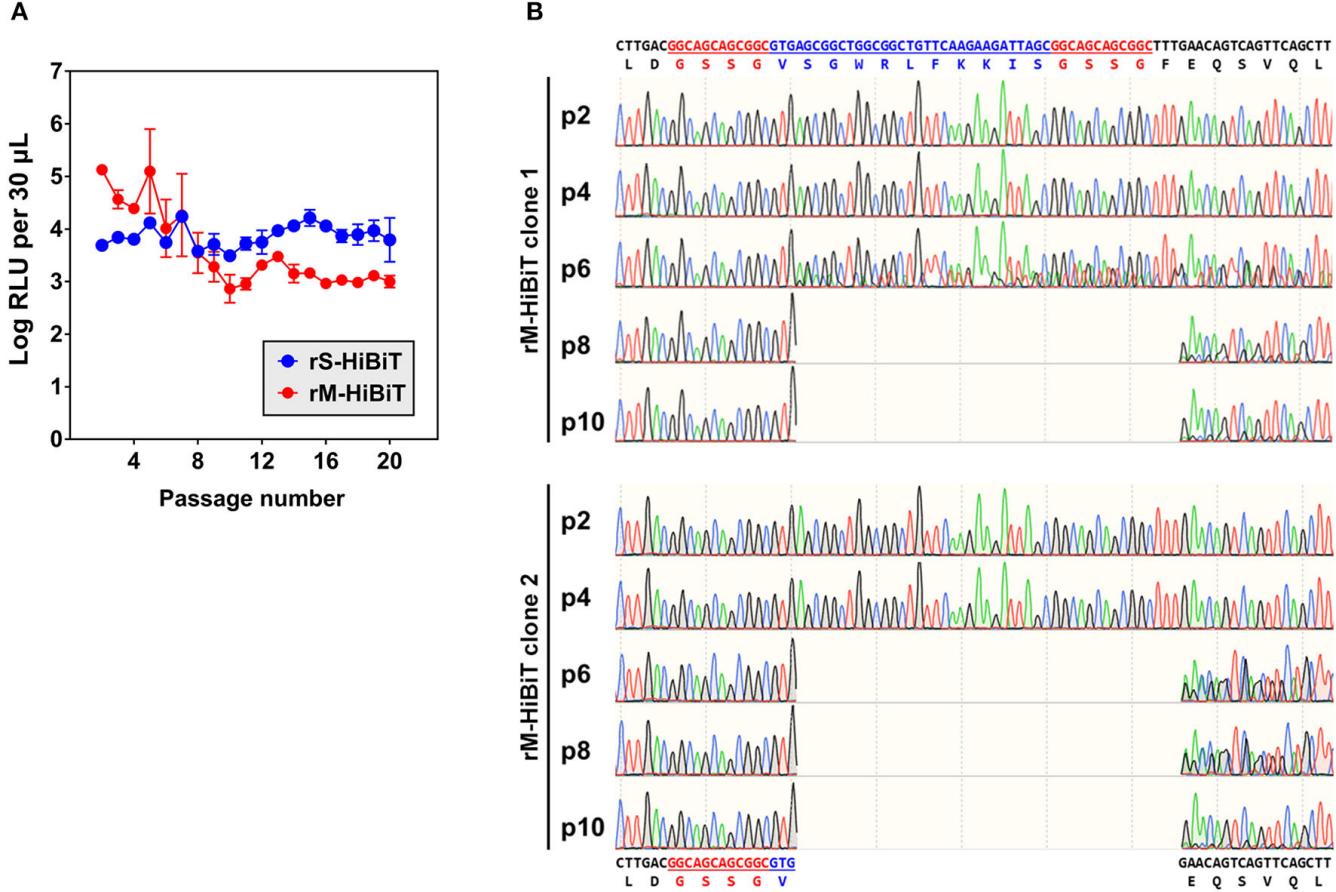
Genetic stability of HiBiT-tagged rIBVs. **(A)** Expression of HiBiT-tagged proteins in passaged rIBVs. The rS-HiBiT and rM-HiBiT were serially passaged at MOI~0.1 in Vero cells for 20 times. Two clones were used for each virus. For each passage, cell lysate samples were subjected to luminescent analysis as in 3C and luminescent intensities were expressed in the unit of log RLU per 30 μL. The average values and standard deviations of the two clones were shown. **(B)** Genetic instability of rM-HiBiT during its passaged in Vero cells. The total RNA of Vero cells infected with rM-HiBiT clone 1 and clone 2 in passage 2, 4, 6, 8, and 10 in **(A)** were extracted and the IBV M gene was amplified by RT-PCR and sequenced. The nucleotide and amino acid sequences of the original HiBiT-M protein and its truncated mutant were shown on the top and at the bottom, respectively. The GSSG linkers were in red and the HiBiT tag was in blue.

To determine the genetic stability of rS-HiBiT and rM-HiBiT, two clones for each rIBV were serially passaged in Vero cells at MOI ~ 0.1 for 20 times, and cell lysates luminescence was determined at each generation. As shown in Figure 4A, the luminescence of rS-HiBiT remained relatively stable through 20 passages with small fluctuations. In contrast, although the luminescence of rM-HiBiT was higher than that of rS-HiBiT in the first 4 passages, it rapidly dropped by 2-log during passage 5–10 and remained low for the remaining generations. Because two clones were passaged, the luminescent instability of rM-HiBiT was unlikely to be a random event, and is possibly due to its intrinsic genetic instability.

To see whether the HiBiT tag was lost during the passage of rM-HiBiT, the M gene was sequenced for passage 2, 4, 6, 8, and 10 for both passaged clones. As shown in Figure 4B, the HiBiT tag was intact in p2 and p4 of rM-HiBiT clone 1. At passage 6, mixed sequences were observed after the first amino acid of the HiBiT tag (Val), but the “base-call” sequence still aligned with the correct sequence. At passage 8 and 10, the “base-call” sequence represented a truncated protein, in which the coding sequence starting from the second amino acid (Ser) of the HiBiT tag to Phe11 of IBV M was deleted. The sequence of the original rM-HiBiT genotype was still recognized as minor peaks downstream of the truncated region. Thus, starting from passage 8, the virus was indeed a mixture of rM-HiBiT and a non-tagged mutant. As for rM-HiBiT clone 2, mixed genotypes were already established starting from passaged 6, and the deletion in the truncated mutant was identical to that of clone 1 (Figure 4B). Notably, the emergence of this non-tagged mutant was temporally consistent with the sudden drop of rM-HiBiT luminescence in Figure 4A. In contrast, in both rS-HiBiT clones passaged, the inserted HiBiT coding sequence and the sequence of its flanking region remained unchanged at passage 20 (data not shown). Taken together, the result suggests that HiBiT luminescence of rS-HiBiT was stable in cell culture for up to 20 passages, whereas that of rM-HiBiT was only stable for 4 passages.

### Quantification of IBV Replication in Developing Chicken Embryos

**Figure 5:**
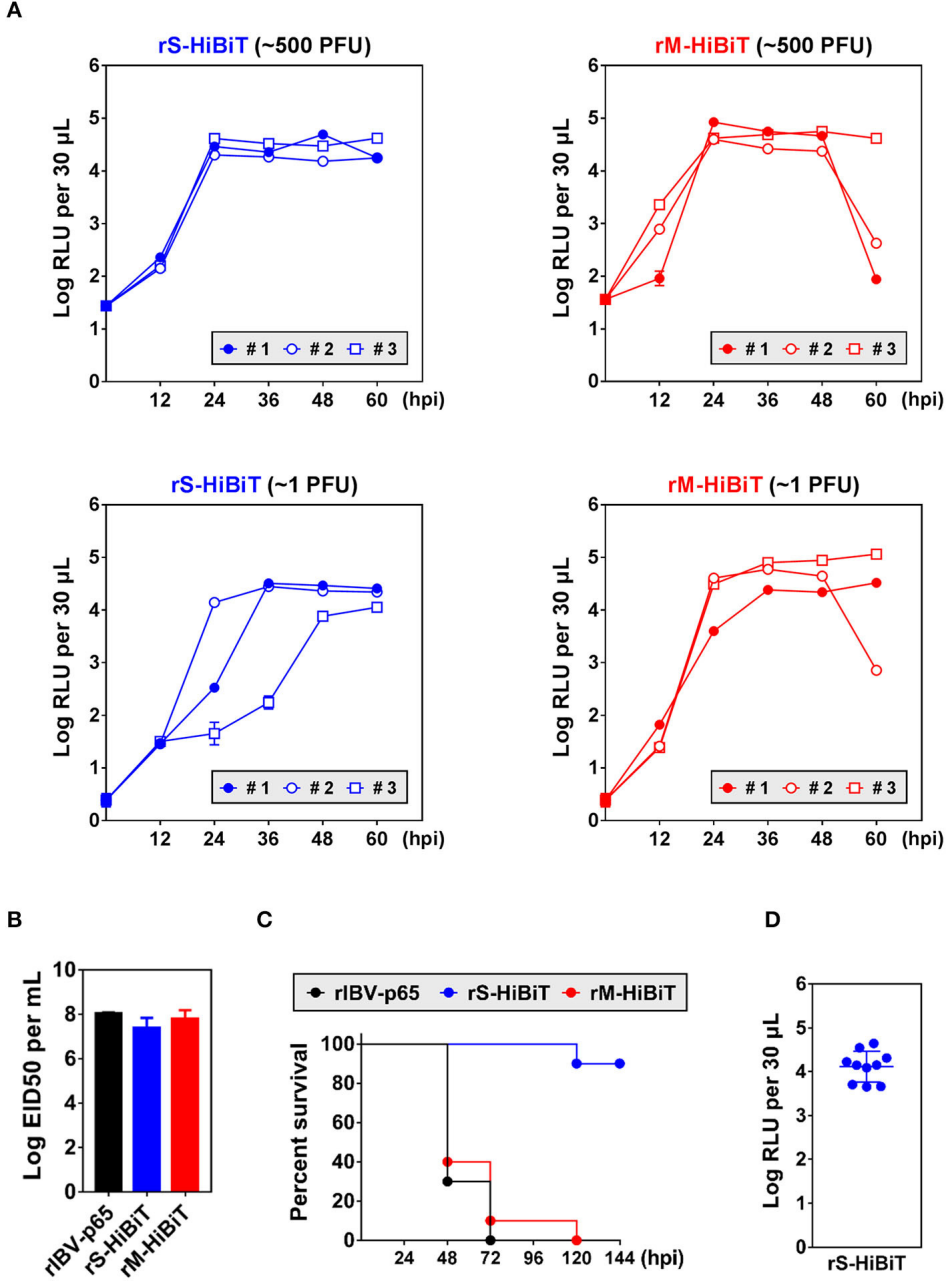
Growth kinetics and attenuation of HiBiT-tagged rIBVs in embryonated chicken eggs. **(A)** Quantification of HiBiT protein in embryonated chicken eggs infected with HiBiT-tagged rIBVs. 10-days-old specific pathogen-free (SPF) embryonated chicken eggs were inoculated with about 500 or 1 plaque forming unit (PFU) of rS-HiBiT or rM-HiBiT. 100 μL allantoic fluid was extracted every 12 h. The background subtracted HiBiT luminescence was expressed in the unit of log RLU per 30 μL. Three eggs (#1, #2, and #3) were used in each set. The experiment was repeated twice with similar results, and the result of one representative experiment is shown. **(B)** The 50% embryo infection doses (EID50) of the three rIBVs. Virus stocks of the rIBV-p65, rS-HiBiT, or rM-HiBiT were 10-fold serially diluted and 0.2 ml diluted virus samples were injected into the allantoic cavities of 10-days old embryonated SPF chicken eggs. The eggs were incubated at 37°C for 5 days. The numbers of infected and uninfected embryos were determined and the EID50 was calculated. The bar chart shows the average results from three independent experiments with standard deviations. **(C)** Survival of the three rIBVs in chicken embryos. About 500 PFU of rIBV-p65, rS-HiBiT, or rM-HiBiT were injected into the allantoic cavities of 10-days old embryonated SPF chicken eggs. Ten eggs were used in each set. The embryos were incubated for 6 days and observed daily for signs of dead embryos. The percentage of survival was plotted against time. The experiment was repeated twice with similar results, and the result of one representative experiment is shown. **(D)** Expression of HiBiT-S protein in rS-HiBiT-infected chicken embryos at 144 hpi in **(C)**. The allantoic fluid was extracted and HiBiT luminescence was determined as in **(A)**.

To see whether HiBiT-tagged rIBVs could be used to quantify viral replication *in vivo*, chicken embryos were inoculated with ~500 or 1 plaque forming unit (PFU) of rS-HiBiT or rM-HiBiT. Low levels of luminescence coming from the inoculated virions were detected at 0 hpi. In embryos infected with ~500 PFU rS-HiBiT, the luminescence of allantoic fluid rapidly increased by more than 3-log during the first 24 h of incubation and remained stable for the next 36 h (Figure 5A). The growth kinetics were very similar among the three infected embryos. In the 1 PFU rS-HiBiT set, the increase of allantoic luminescence was slightly delayed, although peak values were similar to the 500 PFU rS-HiBiT set. Also, there were considerable variations in the growth kinetics among the three embryos, presumably due to the high dilution factor of the inoculated viruses. The allantoic fluid luminescence of embryos infected with rM-HiBiT also reached a high level at 24 hpi and stabilized for the next 24 h (Figure 5A). Notably, in two embryos infected with ~500 PFU and one with ~1 PFU rM-HiBiT, RLU values dropped substantially at 60 hpi. These embryos presumably died from the infection as visible blood vessels were absent.

To see whether the insertion of HiBiT tag affected the *in vivo* replication of IBV, chicken embryos were inoculated with the three rIBVs and the of 50% embryo infection doses (EID50) were determined. EID50 of rIBV-p65 and rM-HiBiT were about 10^8^ per ml (Figure 5B). Although EID50 of rS-HiBiT was slightly lower, the difference was not statistically significant.

To see whether rS-HiBiT and rM-HiBiT were attenuated *in vivo*, chicken embryos were inoculated with ~500 PFU of the three rIBVs and survival rates were monitored. As shown in Figure 5C, 90–100% embryos infected with rM-HiBiT or rIBV-p65 died between day 2 and day 3. In sharp contrast, 90% of the rS-HiBiT-infected embryos survived till day 6. To confirm that rS-HiBiT indeed replicated in these embryos, allantoic fluid luminescence was determined at the end of day 6, and all ten embryos had high RLU values (Figure 5D). To conclude, replication of rS-HiBiT and rM-HiBiT can be monitored continuously in the infected chicken embryos, and rS-HiBiT seems to be attenuated in chicken embryos.

## Discussion

The incorporation of heterologous genes or epitope tags into the CoV genome can greatly facilitate the mechanistic study of viral replication and CoV-host interaction. However, the insertion of whole reporter proteins often resulted in non-viable or highly attenuated recombinant CoVs (Sola et al., 2003; Shen et al., 2009; Hagemeijer et al., 2011; Freeman et al., 2014). In this study, we have successfully recovered two rIBVs, rS-HiBiT and rM-HiBiT, with a HiBiT tag inserted in the respective structural protein. Our data demonstrated that for both rIBV, the HiBiT-tagged viral proteins were effectively expressed and viral replication could be determined in both cell culture and embryonated chicken eggs by chemoluminescent assay. Importantly, rS-HiBiT replicated almost identically as the parental rIBV-p65 and was genetically stable up to passage 20. In short, we have established a sensitive, efficient, and versatile method to quantify IBV replication both *in vitro* and *in vivo*.

Recombinant CoVs and replicons expressing heterologous genes have been generated previously. For example, when a TRS-containing EGFP expression cassette was inserted after the M or N gene, or when the EGFP was fused to the 3′ end of the S gene, infectious rIBVs expressing EGFP could be recovered (Shen et al., 2009). However, these rIBVs were not genetically stable, and the EGFP gene was rapidly lost within 5 passages. As for the replicase gene, GFP-tagging of MHV nsp3 resulted in significant virus attenuation, whereas GFP-tagging of MHV nsp4 was lethal (Hagemeijer et al., 2011; Venkatagopalan et al., 2015). For MHV, the cleavage site between nsp1 and nsp2 seemed to be the only location tolerating the insertion of heterologous genes (Hagemeijer et al., 2011; Tan et al., 2018). By inserting TRS-containing Rluc or Fluc expression cassette into the MHV and FIPV genome, de Haan et al. (2003, 2005) have systematically investigated the effect of genomic position and foreign sequence on the expression levels of heterologous genes and the genetic stability of the recombinant viruses. It was shown that insertions closer to the 3′ end of the genome increased foreign gene expression (de Haan et al., 2003), and the Rluc gene was intrinsically more stably maintained than the Fluc gene (de Haan et al., 2005).

In general, the insertion of short epitope tags in recombinant CoVs was better tolerated. For instance, recombinant CoVs with epitope tags fused with structural, non-structural, or accessory proteins have been described (Yamada and Liu, 2009; Venkatagopalan et al., 2015; Athmer et al., 2017; Florek et al., 2017; Kaewborisuth et al., 2018; Tan et al., 2018). These traditional tags (such as FLAG, HA, Myc) have facilitated the study of protein processing, protein-protein interaction, subcellular localization, membrane topology and so on. However, the detection of epitope-tagged viral proteins often requires additional biochemical assays and quantification is usually semi-quantitative. On the other hand, luciferase-based reporters have clear advantages, such as quantitative measurement, low background and high sensitivity. In fact, using Fluc-expressing recombinant MHVs, it was possible to monitor the spatial and temporal progression of MHV infection in mice by bioluminescence imaging (Raaben et al., 2009). However, the Fluc, Rluc, and Nluc gene are 1,653, 936, and 516 bp in size, respectively, substantially larger than ordinary epitope tags. Therefore, the insertion of whole luciferase genes was not as flexible as that of epitope tags.

The HiBiT system presented in this study has several clear advantages. First, the HiBiT tag is composed of only 11 amino acids, comparable to traditional epitope tags, such as HA and FLAG (9 and 8 amino acids, respectively). The small size means its insertion is less likely to disrupt the structure and function of viral proteins. Second, when reconstituted with the LgBiT subunit, the amount of HiBiT-tagged proteins can be quantitatively determined with high sensitivity. In both cell lysates and supernatant samples, significant luminescence can be detected with a small sample size (30 μL), even at the early stage of infection. The small sample requirement also enabled us to monitor viral growth within a single infected embryo. Third, compared with the time-consuming immunoblot used to detect epitope tags, HiBiT luminescent assay can be finished in 15–20 min without prior sample preparation. Finally, the high affinity between HiBiT and LgBiT also allows the blotting of resolved proteins with high specificity.

Before the development of HiBiT tag, Burkard et al. (2014) have adopted the minimal complementation of β-galatosidase to study the entry of coronavirus and vesicular stomatitis virus (VSV). In this system, the α-peptide of the lacZ β-galactosidase was inserted to the C-terminus of MHV S protein (MHV-Sα), the N-terminus of MHV N protein (MHV-αN), or the C-terminus of VSV G protein (VSV-G) (Burkard et al., 2014). By complementing with the β-galactosidase truncated mutant ΔM15 expressed in the target cells or supplemented in the lysis buffer, active β-galactosidase was reconstituted and virus binding, internalization, and penetration could be quantified using luminescent or fluorescent assays (Burkard et al., 2014). Compared with this LacZα-ΔM15 system, the HiBiT tagging approach has at least two improvements. First of all, the HiBiT tag contains only 11 amino acids, considerably shorter than α-peptide that contains 45 amino acids. Whereas the genetic stability of α-peptide tagged MHV was not determined (passage 2 stocks were used in the study), rS-HiBiT was stable for up to 20 passages. Due to its small size, it is quite likely that genomic locations allowing for viable and stable tag insertion would also be more flexible for HiBiT. Second, luminescent quantification for the LacZα-ΔM15 system is based on the Beta-Glo assay. In this assay, luciferin-galactoside substrate is cleaved by β-galactosidase to form luciferin, which is utilized by a firefly luciferase to generate light. Although the ΔM15 subunit can be expressed by the target cells, the luciferase-gatactoside substrate and the luciferase enzyme are not readily cell-permeable. Therefore, cells have to be lysed at specific time points for luminescence measurement. On the other hand, functional luciferase is directly reconstituted when HiBiT-tagged viral proteins associate with the LgBiT subunit, and permeable extended live cell substrates (such as Endurazine™ and Vivazine™) are also commercially available. Therefore, using cells stably expressing LgBiT, it is possible to perform real-time luminescence quantification. Unfortunately, we were not able to attempt this experiment in the current study due to the lack of essential equipment.

One caveat of fusing the HiBiT tag to structural proteins is the detection of non-infectious virus-like particles (VLPs), defective particles, or extracellular vesicles (EVs). Previous studies have shown that for MHV and IBV, coexpression of the M and E proteins alone resulted in the nucleocapsid-independent assembly of VLPs, which were less dense and sedimented slightly more slowly, compared with virions in sucrose velocity gradients (Vennema et al., 1996; Corse and Machamer, 2000). The M protein of SARS-CoV could also readily self-assemble to form VLPs, either alone or in association with the N protein (Tseng et al., 2010). Also, when expressed using recombinant baculovirus in insect cells, IBV M and S proteins alone were sufficient to assemble into VLPs (Liu et al., 2013). Importantly, spikeless particles and free viral envelopes containing the M protein could also be observed in cryo-electron tomography of MHV (Neuman et al., 2011). Although these VLPs and defective particles are non-infectious, they are secreted into the supernatant along with infectious virions. Similar considerations also apply to the EVs released during coronavirus infection. EVs are lipid bilayer-enclosed structures released by cells into the extracellular environment, which can be divided into three subgroups: exosomes (30–150 nm), microvesicles (50–1,000 nm) and apoptotic bodies (50 nm to 5 μm) (Giannessi et al., 2020). It has been shown that infections caused by HIV and HCV induce the release of EVs to create a proviral environment, but the relationship between coronaviruses and EVs is not fully characterized (Giannessi et al., 2020). In one study, overexpressed SARS-CoV S protein was not readily detected in the pelleted exosomes, unless the transmembrane and cytoplasmic domains were replaced by those of VSV-G (Kuate et al., 2007). On the other hand, because IBV infection induces apoptosis, it is very likely that the released apoptotic bodies contain an abundant amount of viral proteins (Li et al., 2007). Because the HiBiT luminescence assay measures the amount of HiBiT-tagged IBV M or S protein, it cannot differentiate signals from infectious virons and those derived from non-infectious VLPs and/or EVs. In theory, some of the VLPs can be separated from infectious virions using density gradient ultracentrifugation. But due to their remarkable resemblance, there is no reliable method to completely separate infectious particles from contaminating VLPs and EVs (Giannessi et al., 2020). Therefore, the supernatant RLU value measured in this study only serves as a quick surrogate readout of the IBV replication level, and the number of infectious particles has to be determined by assays like TCID50 or plaque assay.

In this study, we have only attempted two insertion sites for the HiBiT tag. Several criteria were taken into consideration. First, only structural proteins were considered because we intended to quantify the amount of released virions by luminescent assays. Secondly, the insertion must not affect trafficking and maturation of the structural protein. For example, the N-terminal signal peptide of S protein must remain intact. Thirdly, to facilitate maximal binding between the HiBiT tag and the LgBiT subunit, the HiBiT tag should be inserted where steric hindrance from the structural proteins was minimal, thus ideally at or near the N-terminus or C-terminus. For the same reason, the ectodomains of transmembrane structural proteins were more suitable, because they are exposed on the exterior side of the virion. For rS-HiBiT, the HiBiT tag was inserted after the S1/S2 cleavage site, also because in our previous study the insertion of a FLAG tag at this location resulted in a stable rIBV that replicated similarly as the rIBV-p65 control, whereas rIBV with a FLAG tag fused to the C-terminus of the S protein replicated considerably more slowly (Yamada and Liu, 2009). In the IBV genome, the coding sequence of the first 10 amino acids of the M gene overlaps with the preceding E gene. To avoid modifying the E gene, the HiBiT tag was therefore inserted between Asp10 and Phe11 of the M protein in rM-HiBiT. We didn't attempt tagging the E protein, because epitope tagging was shown to alter the membrane topology and localization of the IBV E protein (Ruch and Machamer, 2012). Also, in a previous study, the addition of a TC-tag as short as six amino acids (CCPGCC) to the C-terminus of the MHV E protein reduced the peak titer by ~100-fold compared with the parental control (Venkatagopalan et al., 2015). Unfortunately, we were not able to recover rIBV with HiBiT tag inserted at either the N-terminus or C-terminus of the N protein, presumably because such mutations disrupt the normal functions of the N protein, such as genome encapsidation. Given the successful recovery of rS-HiBiT and rM-HiBiT in this study, we will further explore the potential of this system. For example, HiBiT tagging of the non-structural proteins and accessory proteins will be attempted. Also, based on the recently published structure of IBV S protein (Shang et al., 2018), HiBiT tag will be inserted in structurally unhindered locations, such as the loops of the relatively exposed S1-NTD.

Nevertheless, some of the experimental data suggested that the insertion of the HiBiT tag might be accompanied by a loss of viral fitness. First of all, both rS-HiBiT and rM-HiBiT produce plaques that are significantly smaller than the rIBV-p65 control. The small plaque phenotype of rM-HiBiT could be readily explained by its slower growth kinetics. On the other hand, because the growth kinetics of rS-HiBiT was comparable to rIBV-p65, the small plaque phenotype might be attributed to a deficiency in the spreading of progeny virions to neighboring cells and/or the ability to induce cell-cell fusion. In theory, each viral plaque originates from a single infected cell. Restricted by the agarose overlay, viral infection can only be propagated horizontally by infecting or fusing with neighboring cells. This is different from one-step growth curve, where high MOI is used to ensure almost every cell is nearly simultaneously infected. In fact, it is not uncommon for mutant coronaviruses with small plaque phenotypes to exhibit comparable growth kinetics as the controls. For example, recombinant SARS-CoVs and IBVs lacking the E protein ion channel activity formed small plaques but replicated similarly as their corresponding controls in one-step growth curve experiments (Nieto-Torres et al., 2014; Li et al., 2019).

In addition, rS-HiBiT was also significantly attenuated in the infected chicken embryos, as indicated by a much lower mortality rate compared with the rIBV-p65 control. Proteolytic cleavage at the S1/S2 site and S2′ site is an important trigger for coronavirus S proteins to fuse membranes (Li, 2016). Host proteases that cleave S protein include cell surface proteases, lysosomal proteases, proprotein convertases, and extracellular proteases (Li, 2016). Previously, we have shown that the proprotein convertase furin is responsible for cleavage of IBV S protein and determines the susceptibility of IBV in cultured cells (Yamada and Liu, 2009; Tay et al., 2012). Furin is a serine endoproteinase that cleaves the multibasic motif R-X-(R/K/X)-R↓, but the precise cleavage preferences of furin substrates are also affected by flanking amino acid sequences (Remacle et al., 2008). In this study, the insertion of GSSG-HiBiT-GSSG immediately after S538 changed the amino acid sequence downstream of the S1/S2 cleavage site starting from the P2′ position, from RRFRR↓SITEN to RRFRR↓SGSSG. In fact, previous studies have shown that furin and related proprotein convertases prefer aliphatic amino acids (such as Val, Ala, Leu, Ile) at the P2′ position, and the presence of Gly at P2′ decreases the proteolysis efficiency of natural substrates (Remacle et al., 2008). Although the cleavage of S protein is comparable in Vero cells infected with rS-HiBiT or rIBV-p65, IBV S protein might be cleaved by different host protease (s) in the infected chicken embryos, and the abundance and activity of proteases from different host species may also vary (Li, 2016). Therefore, it is possible that reduced S protein processing of rS-HiBiT affected its propagation in the chicken embryos, thereby leading to an attenuated phenotype.

The rapid elimination of the HiBiT tag in rM-HiBiT suggested that there was a strong selection pressure against the insertion of foreign sequences between D10 and F11 in the ectodomain of the IBV M protein. As the same deletion was observed in two independent passaged rM-HiBiT clones, it is possible that the insertion of HiBiT in this position resulted in a hotspot for recombination, and longer or shorter deletions were lethal. In one early study using the VLP formation assay, mutant MHV M protein with an internal deletion from A7 to F22 in the ectodomain was not secreted, suggesting that the deleted region in the ectodomain might be essential for virion assembly (de Haan et al., 1998). As for SARS-CoV, neither the N-linked glycosylation blocking mutation N4Q nor alanine substitutions of the di-leucine motif L15–L16 affected SARS-CoV M secretion or VLP assembly, although other residues in the ectodomain were not investigated (Tseng et al., 2013). Importantly, because of the homotypic interactions between CoV M proteins at multiple contact sites, mutant MHV M proteins that were not able to assemble into VLPs by themselves could still associate with assembly-competent M proteins and thereby co-incorporated into VLPs (de Haan et al., 2000). This may explain the genetic instability and yet persistence of rM-HiBiT observed in this study. The HiBiT tag inserted between D10 and F11 might have a deleterious effect on virion assembly. The partial deletion mutant (D10-GSSGV-F11) emerged after passage 6 might have partially restored this defect, allowing the coexisting rM-HiBiT to efficiently assemble and persist in the mixed population.

To conclude, we have rescued and characterized two HiBiT-tagged rIBVs and demonstrated the advantages of using this method for efficient *in vitro* and *in vivo* viral quantification. Together with the various animal models for human CoVs, HiBiT-tagged recombinant CoVs may facilitate the mechanistic study of CoV replication and pathogenesis.

## Materials and Methods

### Construction of Recombinant IBV

The egg-adapted Beaudette strain of IBV (ATCC VR-22) was obtained from American Type Culture Collection (ATCC) and adapted to Vero cells as previously described (Fang et al., 2005). This Vero-adapted strain was named IBV-p65, and the complete genome sequence was uploaded (accession no. DQ001339) (Fang et al., 2005). The infectious cDNA clone of IBV-p65 was constructed, and the recovery of recombinant IBV-p65 (rIBV-p65) was described previously (Fang et al., 2007). Briefly, genome fragments were amplified by RT-PCR from total RNA extracted from IBV-p65-infected Vero cells and cloned to plasmid vectors. The five plasmids (and the genomic regions) were pKT-p65-F1 (1–5,752 nt), pGEM-p65-F2 (5,749–8,693 nt), pCR-XL-p65-F3 (8,690–15,528 nt), pGEM-p65-F4 (15,525–20,900 nt), and pGEM-p65-F5 (20,897–27,614 nt). A T7 promoter was added to the 5′ end of F1 fragment, and a polyadenylate (A32) sequence was added to the 3′ end of F5 fragment. Flanked by Type IIS restriction enzyme sites, the five fragments were prepared by digestion of the corresponding plasmids with either Esp3I (NEB, R0734) or BsaI (NEB, R3733), followed by gel extraction of respective bands. The full-length cDNA clone was seamlessly assembled from the purified fragments using T4 DNA ligase (NEB, M0202M). The ligation product was precipitated and served as the template for *in vitro* transcription using the mMessage mMachine T7 kit (Ambion). The N transcript was generated from a linearized pKT-IBVN construct containing the IBV N gene and the 3′-UTR region. The full-length transcript and the N transcript were introduced into Vero cells using Gene Pulser X-cell electroporation system (Bio-Rad) at 100 V square wave for 25 ms. Electroporated cells were incubated in DMEM supplemented with 1% FBS at 37°C overnight. The medium was then changed to serum-free DMEM, and the cells were incubated for 72–96 h and monitored for the appearance of CPE. Recombinant viruses were harvested by three freeze-thaw cycles and purified by two rounds of plaque purification.

To construct rS-HiBiT, the genomic region 21,949–22,227 nt of rIBV-p65 was synthesized by Genewiz (Hangzhou, China), with the following sequence *GGCAGCAGCGGC*GTGAGCGGCTGGCGGCTGTTCAAGAAGATTAGC*GGCAGCAGCGGC* inserted between T21981 and A21982. This 57 bp sequence encoded the HiBiT tag (VSGWRLFKKIS) and the linker sequence (*Gly-Ser-Ser-Gly*) on both sides, and was inserted between the codons of Ser538 and Ile539 of the S gene. Using this synthesized plasmid as template, the insert fragment was amplified with forward primer ATCACTAATGGAACACGTCG and reverse primer ATCCAGAGAACTGCCACAAA. The vector fragment was amplified using pGEM-p65-F5 as template with forward primer CGACGTGTTCCATTAGTGAT and reverse primer TTTGTGGCAGTTCTCTGGAT. The insert and vector was joined using Vazyme ClonExpress II One Step Cloning Kit (Nanjing, China), resulting in the plasmid pGEM-p65-F5-rS-HiBiT. This plasmid was used in place of pGEM-p65-F5 to recover rS-HiBiT, using the same procedures for rIBV-p65.

To construct rM-HiBiT, insert 1 was amplified from pGEM-p65-F5 using forward primer GGGAAATAGAGTCAGCTG and reverse primer TTCTTGAACAGCCGCCAGCCGCTCACGCCGCTGCTGCCGTCAAGAGTACAATTTGTCTCG. Insert 2 was amplified from pGEM-p65-F5 using forward primer CTGGCGGCTGTTCAAGAAGATTAGCGGCAGCAGCGGCTTTGAACAGTCAGTTCAGC and reverse primer TATGCGCTCTTAAAACAGAG. The vector fragment was amplified from pGEM-p65-F5 using forward primer CTCTGTTTTAAGAGCGCATA and reverse primer CAGCTGACTCTATTTCCC. Insert 1, insert 2, and vector were joined using Vazyme ClonExpress II One Step Cloning Kit (Nanjing, China), resulting in the plasmid pGEM-p65-F5-rM-HiBiT. In this plasmid, the 57 bp linker-HiBiT-linker sequence, identical to that in rS-HiBiT, was inserted between C24535 and T24536 of the original rIBV-p65 genome, between the codons of Asp10 and Phe11 in the M gene. This plasmid was used in place of pGEM-p65-F5 to recover rM-HiBiT, using the same procedures for rIBV-p65. The genotypes of both rS-HiBiT and rM-HiBiT were validated by sequencing as described below.

### Cell Culture and Virus Infection

Vero, DF1, and BHK cells were cultured in DMEM supplemented with 5% FBS and 1% Penicillin-Streptomycin (Gibco). H1299 cells were cultured in RPMI1640 medium (Gibco) supplemented with 5% fetal bovine serum (FBS) and 1% Penicillin-Streptomycin (Gibco). All cells were grown in a 37°C incubator supplied with 5% CO_2_.

To prepare the virus stocks, monolayers of Vero cells were infected with rIBV-p65, rS-HiBiT, or rM-HiBiT at MOI ~ 0.1 and cultured in plain Dulbecco Modified Eagle Medium (DMEM, Gibco) at 37°C until complete fusion of the entire monolayer was observed. After three freeze/thaw cycles, cell lysate was clarified by centrifugation at 1,500 g at 4°C for 30 min. The supernatant was aliquot and stored at −80°C as the virus stock. The titer of the virus stock was determined by plaque assays. For all three rIBVs, passage 2 virus stocks were used for all the experiments. The mock lysate was prepared by the same treatment of uninfected Vero cells.

Unless stated otherwise, for IBV infection experiments in cultured cells, cells seeded on 12-well plate were first washed twice with serum-free medium. The cells were then infected with IBV at MOI ~ 2 or incubated with an equal volume of mock lysate. After 2 h of adsorption, the cells were washed twice and incubated in serum-free medium at 37°C until they are harvested. Phase images of IBV-infected cells were captured using the Nikon ECLIPSE Ts2 inverted microscope with a 20× objective. Cell lysates and supernatant samples were harvested as stated below.

### Antibodies/Antisera and Embryonated Chicken Eggs

The antibody against β-actin (#4967) was purchased from Cell Signaling Technology. The antisera against IBV S, M, and N protein were isolated from rabbits immunized with bacterial expressed fusion proteins as previously described (Liu and Inglis, 1991; Li et al., 2005).

Eight-days-old specific pathogen-free (SPF) embryonated chicken eggs were obtained from the Laboratory Animal Center of South China Agricultural University and incubated at 37°C for 24 h and dead embryos were discarded before infection experiments.

### Virus Titration by Tissue Culture Infective Dose 50 Method

Supernatant samples were harvested from IBV-infected cells and clarified by centrifugation at 16,000 × g at 4°C for 5 min. Cell lysate samples were harvested by subjecting IBV-infected cells to three freeze-thaw cycles and clarified by centrifugation at 16,000 × g at 4°C for 5 min. Virus samples were kept at −80°C for <2 weeks before the titration experiment. Virus titer was determined by the tissue culture infective dose 50 (TCID50) assay. Briefly, virus samples were 10-fold serially diluted. Confluent monolayers of Vero cells seeded on 96-well plates were washed once with plain DMEM, and 100 μl diluted virus sample was added to each well, with 8 wells used for each dilution. Cells were incubated at 37°C for 3–5 days and examined with a phase-contrast microscope. Wells were determined as either positive (with CPE) or negative (without CPE), and TCID50 was calculated using the Reed and Muench method (Reed and Muench, 1938). The virus titer was expressed in the unit of the logarithm of TCID50 per ml. Each sample was titrated in duplicate or triplicate in each experiment.

### Plaque Assay and Plaque Size Measurement

Virus stocks of the three rIBVs were 10-fold serially diluted using serum-free medium. Confluent monolayers of Vero cells seeded on 6-well plates were washed once with plain DMEM, and 200 μl diluted virus sample was added to each well. The plates were agitated every 10–15 min to ensure proper coverage. After 2 h of adsorption, unbound viruses were removed and cells were washed once with plain DMEM. Two milliliters overlay medium (0.4% agarose in DMEM) was added to each well and the plates were incubated at 37°C for 2 days before plaques formed. Agarose overlay was removed and cells were fixed with 4% formaldehyde before staining with crystal violet. The plates were then scanned as 8-bit grayscale images and the areas of plaques were determined using the ImageJ software. The areas of at least 100 plaques were determined for each recombinant virus.

### SDS-PAGE and Western Blot Analysis

To obtain whole-cell lysates for protein analysis, cells were harvested at the indicated time points using cell scrapers (Corning) and collected by centrifugation at 16,000 × *g* for 1 min. The supernatant was discarded, and the cell pellet was lysed in 1 × RIPA buffer (10 mM Tris-HCl pH 8.0, 140 mM NaCl, 0.1% SDS, 1% Triton X-100, 0.1% sodium deoxycholate, 1 mM EDTA, and 0.5 mM EGTA). After clarified by centrifugation, the protein concentration of the cell lysate was determined. The cell lysate was then mixed with 5× Laemmli sample buffer (0.3125 M Tris-HCl pH 6.8, 10% SDS, 50% glycerol, 25% β-mercaptoethanol, and 0.025% bromphenol blue), boiled at 90°C for 5 min, and centrifuged at 16,000 × g for 5 min (Laemmli, 1970). Equal amounts of protein samples were loaded to each well and separated by sodium dodecyl sulfate-polyacrylamide gel electrophoresis (SDS-PAGE) using the Bio-Rad Mini-PROTEAN Tetra cell system. The resolved proteins were then transferred to a 0.2 μm nitrocellulose membrane using the Bio-Rad Trans-Blot protein transfer system. To block off non-specific binding sites, the membrane was incubated with 5% skim milk in 1 × TBST buffer (20 mM Tris-HCl pH 7.4, 150 mM NaCl, 0.1% Tween 20) at room temperature for 1 h. The membrane was then incubated with 1 μg/ml specific primary antibody dissolved in 1 × TBST with 3% BSA (w/v) at 4°C overnight. The membrane was washed three times with 1 × TBST, and incubated with 1:10000 diluted IRDye 800CW Goat anti-Rabbit or 680RD goat anti-mouse IgG secondary antibodies (Licor) at room temperature for 2 h. The membrane was washed three times with 1 × TBST, and fluorescence imaging was performed using the Azure c600 Imager according to the manufacturer's instruction. All experiments were repeated for at least three times with similar results, and one of the representative results was shown.

### Determination of HiBiT-Tagged Proteins by HiBiT Blotting

Cell lysates were resolved by SDS-PAGE and transferred to a 0.2 μm nitrocellulose membrane as described above. The HiBiT-tagged proteins were detected using the Nano-Glo HiBiT blotting system (Promega). Briefly, 50 μL of LgBiT protein was mixed with 10 ml blotting buffer to prepare the LgBiT/buffer solution. The membrane was rinsed with 1 × TBST once and incubated in the LgBiT/buffer solution at room temperature for 1 h. Next, 20 μL of substrate solution was added to the LgBiT/buffer solution, and the membrane was incubated for 5 min. The chemoluminescence of the HiBiT-tagged protein was detected using the Azure c600 Imager according to the manufacturer's instruction. All experiments were repeated for at least three times with similar results, and one of the representative results was shown.

### Quantification of HiBiT-Tagged Proteins in Cell Culture Samples

The expression level of HiBiT-tagged proteins was determined using the Nano-Glo HiBiT lytic detection system (Promega). Briefly, cells seeded on 96-well plate were infected with rIBV at MOI ~ 2. For each timepoint three wells were infected. After 2 h of adsorption, the cells were washed twice and incubated in 150 μL serum-free medium at 37°C until they are harvested. The Nano-Glo HiBiT lytic reagent was prepared by diluting the LgBiT protein 1:100 and the substrate solution 1:50 into an appropriate volume of lytic buffer. At each time point, 50 μL culture supernatant was transferred to a tube and mixed with 50 μL lytic reagent. One hundred microliters lytic reagent was added to the remaining medium in the well. The plate was mixed at 500 rpm on an orbital shaker for 5 min, and all samples were incubated at room temperature for 15–30 min. The chemoluminescence of 60 μL supernatant or cell lysate sample was measured using Synergy H1 hybrid multi-mode microplate reader with an integration time of 2 s. Each sample was assayed in triplicate in each experiment. Readings from uninfected wells were used as the empty background. The background-subtracted luminescence was expressed in the unit of the logarithm of RLU per 30 μL. The plotted data represented the means and standard deviations from three independent experiments.

### Serial Passage and Sequencing of HiBiT-Tagged rIBVs

Vero cells seeded in 35 mm dishes were infected with rS-HiBiT or rM-HiBiT at MOI ~ 0.1, and two clones were used for each virus. Unbound viruses were removed after 2 h and the cells were replaced with 1.5 ml plain DMEM after two rounds of PBS wash. When complete CPE was observed, the dishes were frozen at −80°C. After three freeze/thaw cycle, cell lysate was clarified by centrifugation at 1,500 g at 4°C for 10 min. Ten microliters of the clarified lysate was used to infect Vero cells for the next viral passage. The remaining lysates were subjected to luminescence measurement as described above. For each virus, two clones were passaged for 20 generations. The plotted data represent the means and standard deviations from four samples (two independent experiments, each with two samples).

To sequence the HiBiT inserted region, total RNA was extracted from cell lysates using the TRIzol reagent (Invitrogen) according to the manufacturer's instructions. Briefly, 100 μL cell lysate was added to 0.9 ml TRIzol, and vigorously mixed with 200 μL chloroform. The mixture was then centrifuged at 12,000 × g at 4°C for 15 min, and the aqueous phase was mixed with an equal volume of isopropanol. The RNA was precipitated by centrifugation at 12,000 × g at 4°C for 15 min, washed twice with 70% ethanol, and dissolved in 30 μL RNase-free water. The total RNA was reverse transcribed using the FastKing gDNA Dispelling RT SuperMix kit (Tiangen) according to the manufacturer's instructions. Briefly, 2 μg total RNA was mixed with 4 μL 5 × FastKing-RT SuperMix (containing RT enzyme, RNase inhibitor, random primers, oligo dT primer, dNTP and reaction buffer) in a 20 μL reaction mixture. Using a thermo cycler, reverse transcription was performed at 42°C for 15 min and the RT enzyme was then inactivated at 95°C for 3 min. Using the RT product as the template, the HiBiT flanking regions were amplified by PCR. For rS-HiBiT, forward primer TTACGGTCCTCTTCAAGGTGG and reverse primer ATCCAGAGAACTGCCACAAA were used, and the PCR product was sequenced using the primer TTACGGTCCTCTTCAAGGTGG. For rM-HiBiT, forward primer ACAATCCGGAATTAGAAGCA and reverse primer TATGCGCTCTTAAAACAGAG were used, and the PCR product was sequenced using the primer ACAATCCGGAATTAGAAGCA. The sequencing result were aligned with the expected sequences using the Snapgene software.

### Infection of Chicken Embryos With IBV and Quantification of HiBiT-Tagged Proteins in the Allantoic Fluid

Two hundred microliters PBS-diluted virus solutions containing ~500 PFU or 1 PFU of rS-HiBiT or rM-HiBiT were innoculated into the allantoic sack of 10-days-old chicken embryos. Three embryos were infected in each set. The puncture was sealed with scrotch tape. In the next 60 h, about 100 μL allantoic fluid was extracted every 12 h, the puncture was resealed, and the embryo was returned to the incubator. The collected allantoic fluid was stored at −80°C temporarily. To quantify HiBiT-tagged proteins, 100 μL allantoic fluid was mixed with 100 μL Nano-Glo HiBiT lytic reagent and incubated at room temperature for 15–30 min. For each sample, 60 μL mixture was aliquoted to each of three wells into an opaque white 96-well plate. Readings from uninfected embryos were used as the empty background. The luminescence was measured as describe above. The plotted data point represented the mean and standard deviation of each infected embryo. The experiment was repeated twice with similar results, and the result of one representative experiment is shown.

### Determination of the 50% Embryo Infection Dose

The virus stocks of the three rIBVs were 10-fold serially diluted with DMEM, and 10^3^- to 10^8^-fold diluted viruses were used. Two hundred microliters diluted virus solution was injected into the allantoic cavity of 10-days-old SPF embryonated chicken eggs. Five eggs were used for each dilution. The eggs were incubated at 37°C for 5 days, transferred to a 4°C refrigerator, and incubated overnight. The chicken embryos were extracted and examined for the signs of death or stunted growth. In case of ambiguity, the corresponding allantoic fluid was analyzed by Western blot using anti-IBV N antiserum. The number of infected or uninfected embryos was counted, and EID50 was calculated using the Reed and Muench method (Reed and Muench, 1938). The experiment was repeated three times with similar results, and the mean and standard deviation values of EID50 were plotted.

### Survival Curves of IBV-Infected Chicken Embryos

Two hundred microliters PBS-diluted virus solutions containing ~500 PFU of the rIBV-p65, rS-HiBiT, or rM-HiBiT were innoculated into the allantoic sack of 10-days-old chicken embryos. Ten eggs were infected for each virus. The embryos were incubated for 6 days and observed daily for signs of dead (disappearance and/or detachment of blood vessels). The survival rate was calculated by dividing the number of surviving embryos by the number of total embryos. The experiment was repeated twice with similar results, and the result of one representative experiment is shown.

### Statistical Analysis

The one-way ANOVA method was used to analyze the significant difference between the indicated sample and the respective control sample. Significance levels were presented by the *p*-value (ns, non-significant; ^*^p < 0.05; ^**^p < 0.01; ^****^p < 0.0001).

## Data Availability Statement

The raw data supporting the conclusions of this article will be made available by the authors, without undue reservation.

## Author Contributions

TF and DL designed and organized the study. XL, QZ, and JL performed the experiments. SL performed some of the experiments. TF and DL wrote the paper. All authors contributed to the article and approved the submitted version.

## Conflict of Interest

The authors declare that the research was conducted in the absence of any commercial or financial relationships that could be construed as a potential conflict of interest.
